# The Role of Angiogenesis in Cancer Treatment

**DOI:** 10.3390/biomedicines5020034

**Published:** 2017-06-21

**Authors:** Mehdi Rajabi, Shaker A. Mousa

**Affiliations:** Pharmaceutical Research Institute, Albany College of Pharmacy and Health Sciences, Rensselaer, NY 12144, USA; mehdi.rajabi@acphs.edu

**Keywords:** physiological angiogenesis, pathological angiogenesis, pro-angiogenesis, anti-angiogenesis, endothelial cells, pericyte, integrin, matrix metalloproteinase, vascular growth factors, anti-VEGF, anti-integrin, tyrosine kinase inhibitors

## Abstract

A number of anti-angiogenesis drugs have been FDA-approved and are being used in cancer treatment, and a number of other agents are in different stages of clinical development or in preclinical evaluation. However, pharmacologic anti-angiogenesis strategies that arrest tumor progression might not be enough to eradicate tumors. Decreased anti-angiogenesis activity in single mechanism-based anti-angiogenic strategies is due to the redundancy, multiplicity, and development of compensatory mechanism by which blood vessels are remodeled. Improving anti-angiogenesis drug efficacy will require identification of broad-spectrum anti-angiogenesis targets. These strategies may have novel features, such as increased porosity, and are the result of complex interactions among endothelial cells, extracellular matrix proteins, growth factors, pericyte, and smooth muscle cells. Thus, combinations of anti-angiogenic drugs and other anticancer strategies such as chemotherapy appear essential for optimal outcome in cancer patients. This review will focus on the role of anti-angiogenesis strategies in cancer treatment.

## 1. Introduction

**Figure 1:**
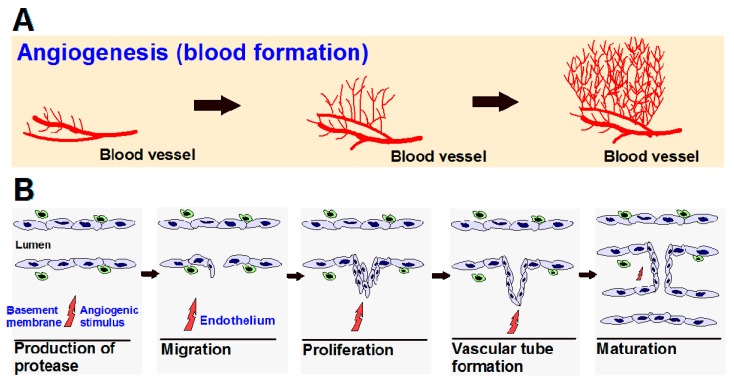
(**A**) Angiogenesis is the process of the development of new blood vessels from pre-existing vessels, which allows for tumor progression; (**B**) Steps in angiogenesis.

Angiogenesis is a normal and complex process controlled by certain biomolecules produced in the body. Endogenous local or systemic chemical signals coordinate functions of endothelial cells and smooth muscle cells to repair damaged blood vessels. The generation of new blood vessels is from pre-existing blood cells via the “sprouting” of endothelial cells, thus expanding the vascular tree (Figure 1A) [1,2]. Steps toward angiogenesis include protease production, endothelial cell migration, and proliferation, vascular tube formation, anastomosis of newly formed tubes, synthesis of a new basement membrane, and incorporation of pericytes and smooth muscle cells (Figure 1B).

After activation of endothelial cells by angiogenic stimuli, proteolytic enzymes are produced, which degrade the perivascular extracellular matrix (ECM) and the basement membrane. Endothelial cells proliferate and migrate into the perivascular area, forming “primary sprouts”. Subsequent lumenation of these primary sprouts leads to formation of capillary loops, which is followed by synthesis of a new basement membrane and blood vessel maturation to complete tube-like structures through which blood can flow [3].

Physiological angiogenesis processes are crucial during embryo development, wound healing, and collateral formation for improved organ perfusion. However, abnormally accelerated angiogenesis processes or pathological angiogenesis are associated with various disorders, including ocular neovascularization, which leads to a loss of vision.

In comparison with chemical signals that induce blood formation, there is another type of chemical signal known as an angiogenesis inhibitor (Table 1). These signals may systematically disrupt blood vessel formation or support removal of existing vessels. Inhibitors function by acting on several proteins that have been identified as angiogenic activators, including vascular endothelial growth factor (VEGF), basic fibroblast growth factor (bFGF, FGF2), angiogenin, transforming growth factor (TGF)-α, TGF-β, tumor necrosis factor (TNF)-α, platelet-derived endothelial growth factor, granulocyte colony-stimulating factor, placental growth factor, interleukin-8 (IL-8), hepatocyte growth factor, and epidermal growth factor [4]. It is very important to keep a balance between activators and inhibitors, and this balance regulates vascular homeostasis.

Among them, VEGF is a powerful angiogenic agent in neoplastic tissues, and VEGF receptors (VEGFR) have been widely studied in the field of neoplastic vascularization. For example, by generation of VEGF and its secretion into neighboring tissue, the tumor cells will be able to feed on the new blood vessels.

Although it was thought for many years that the spread of cancer cells and growth of localized tumors beyond a few millimeters in size requires local angiogenesis in which tumor cells produce new blood vessels by releasing pro-angiogenic chemical signals, recent studies have reported that tumors like brain, lung, and liver can co-opt and grow along existing vessels without evoking new vessel growth [31]. Normal cells proximal to cancer cells may also support a pro-angiogenic response via signaling molecules. Local neovascularization supplies growing tumors with oxygen and essential nutrients, supports tumor extension and invasion into nearby normal tissue, and is essential to distant metastasis [32,33].

## 2. Angiogenesis Mechanism in Cancer

**Figure 2:**
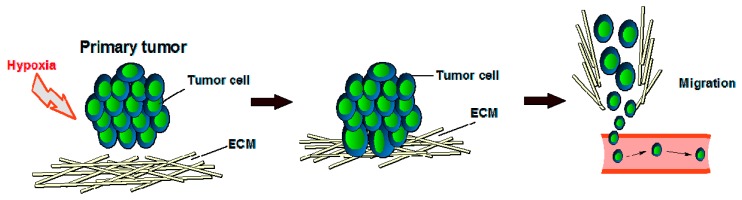
Tumor development under hypoxic conditions. ECM = extracellular matrix.

It is well known that in healthy cells, oxygen tension is key in the regulation of angiogenesis, and endothelial cells (ECs) and smooth muscle cells (SMCs) have various oxygen-sensing mechanisms, including oxygen-sensitive NADPH oxidases, endothelial nitric oxide synthase (eNOS), and heme-oxygenases [34]. Vascular cells also express a different class of oxygen sensors that interface with the hypoxia-inducible transcription factor (HIF) family, which in turn is an important molecular interface for relaying adaptations to changes in oxygen tension. Each of the three isoforms of HIFα (HIF-1–3) can heterodimerize with the aryl hydrocarbon receptor nuclear translocator (HIFβ/ARNT) subunit to form an active transcriptional complex that initiates expression of hundreds of genes, including those regulating cell survival, metabolism, and angiogenesis [35]. In order to grow or locally metastasize, tumor tissue also needs oxygen and nutrients that will be provided by blood vessels [32] because the primary function of blood vessels is to carry the oxygen that we breathe. The presence and abundance of oxygen correlates with the metabolism of endothelial cells in which oxygen can be consumed to form either sprouts in vitro [36] or a vascular network in vivo [37]. Because oxygen is key in cell growth (both healthy cells and cancer cells), hypoxic tumor cells (tumor cells that have been deprived of oxygen) will not divide (Figure 2). In growing cancers, endothelial cells are vigorously active because of the release of many proteins, such as EGF, estrogen, basic and acidic FGF, IL-8, prostaglandin E1 and E2, TNF-α, and VEGF, that can activate endothelial cell growth and motility when the anti-angiogenic factors’ production is reduced [32,38]. VEGF and bFGF are particularly important to tumor angiogenesis [32], but the redundancy of (other) pro-angiogenic factors helps explain the current suboptimal effectiveness in the oncology of the pharmacological inhibitors of single endogenous angiogenic agents.

In comparison to other naturally occurring angiogenesis inhibitors such as angiostatin, endostatin, interferons, IL-1 and IL-12, tissue inhibitor of metalloproteinases, and retinoic acid [38,39,40], we previously reported that physiological concentrations of thyroid hormone are pro-angiogenic by multiple mechanisms. This raises the possibility that thyroid hormone (thyroxine) is a model of non-protein stimulators of angiogenesis that may contribute to clinical resistance to anti-angiogenesis drugs [41,42,43]. We also introduced compound MR-49 as a novel pro-angiogenesis modulator that is synthesized from tetraiodothyroacetic acid (tetrac), a deaminated derivative of thyroxine hormone. MR-49 expressed a pro-angiogenic rather than an anti-angiogenic activity of tetrac [44]. Prostaglandin E2 (PGE2) as a mitogen in epithelial tumor cells is another example of a non-protein stimulator of angiogenesis in the vascular endothelium. It has also been also shown that the overexpression of cyclooxygenase-2 (an enzyme for conversion of arachidonic acid to prostaglandin H2) is accompanied by enhanced expression and production of angiogenic factors such as VEGF, FGF-2, HIF-1, matrix metalloproteinases (MMPs), and adhesion receptors of the integrin families. Therefore, it has been found that, with a high output of PGE2 via expression of cyclooxygenase-2, angiogenesis causes tumor development [45,46]. Furthermore, the CCN family of matricellular proteins are cytokines linking cells to the extracellular matrix. CCN3 is pro-angiogenic, while CCN5 is anti-angiogenic [47,48,49,50]. Multimerin 2 (MMRN2) has anti-angiogenesis effects, and its down-modulation occurs in the context of tumor-associated angiogenesis [51,52].

## 3. Side Effects in Anti-Angiogenic Therapy

It has been reported that angiogenesis inhibitors might potentially interfere with many normal body processes such as wound healing [53], blood pressure [54], kidney function, fetal development, reproduction, and increased risk of clots in the arteries that would result in stroke or heart attack [53,55,56]. As an example, hypertension is one of the most observed side effects of systemic inhibition of VEGF signaling, which is also one of the most manageable side effects with the use of standard anti-hypertensive medications. Treatment of cancer by the inhibition of VEGF signaling will cause endothelial dysfunction by decreasing the level of VEGF, which will eventually result in hypertension.

Under normal conditions, VEGF is known to release vasodilator nitric oxide (NO) in vessel walls by upregulating endothelial nitric oxide synthase (eNOS) and prostacyclin (PGI2), resulting in vasodilation, through the activation of the mitogen-activated protein kinase (MAPK) and phosphatidylinositol 3-kinase (PI3K) downstream pathways [57,58,59,60]. Therefore, by inhibition of VEGF, the production of NO will be decreased, which will promote vasoconstriction, increase the peripheral resistance and eventually elevate blood pressure [61].

Because angiogenesis is required for wound healing, high levels of VEGF are produced during the repairing of normal wound. It has been reported that the inhibition of VEGF in angiogenesis therapy could interfere with normal angiogenesis and cause a disruption in the angiogenesis process or lead to a delay of the wound-healing process. In this regard, the treatment of patients having metastatic colorectal cancer with bevacizumab showed an increase in post-surgical wound healing complications, including wound dehiscence and impaired wound healing [62]. It was thought earlier that anti-angiogenesis strategies by blocking tumor angiogenesis would limit permeability of its own and other adjunct therapies. However, data demonstrated that treatment with anti-angiogenic drugs results in a more efficient normalized vasculature that might allow for improved tumor delivery of drugs [63,64,65,66,67,68,69,70]. Additionally, other anti-angiogenesis strategies such as anti-αvβ3 integrin have been exploited for enhanced active targeted delivery into tumors [71,72].

## 4. Examples of Angiogenesis Inhibitors for Cancer Therapy

Angiogenesis inhibitors can be designed to block the formation of new blood vessels, and the growth of tumors would thereby be halted but not eliminated; hence, anti-angiogenesis monotherapies are not effective in humans as was hoped for [73,74]. Thus, combinatorial treatments with conventional chemotherapy drugs are required. These inhibitors sometimes may not eliminate tumors and in order to achieve optimal treatment, a combination of anti-angiogenesis and conventional chemotherapy may be required.

In general, angiogenesis inhibitors can be classified into two main group of inhibitors: (i) direct inhibitors that target endothelial cells in the growing vasculature, and (ii) indirect inhibitors that target either tumor cells or the other tumor-associated stromal cells [75].

In direct inhibition of angiogenesis, inhibitors such as angiostatin, endostatin, arrestin, canstatin, and tumstatin are known as fragments released on proteolysis of distinct ECM molecules and prevent vascular endothelial cells from proliferating and migrating in response to a spectrum of angiogenesis inducers, including VEGF, bFGF, IL-8, and PDGF [14,76,77,78]. It has also been reported that the direct anti-angiogenic effect can be attributed to integrin receptors accompanied by several intracellular signaling pathways [14]. For example, Eikesdal et al. identified the critical amino acids (L, V, and D) within tumstatin, known as an inhibitor of endothelial cell proliferation, that confer anti-angiogenic and antitumor activity to tumstatin peptide, which is associated with the expression of the adhesion receptor, α_v_β_3_ integrin, on tumor endothelial cells [79].

**Figure 3:**
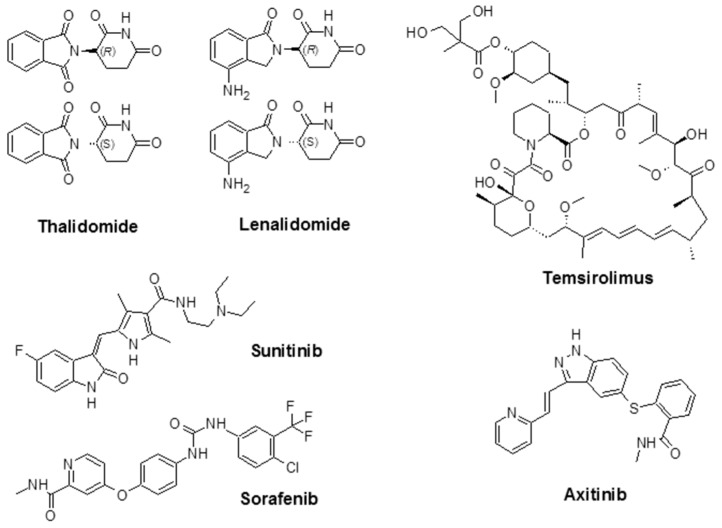
Chemical structure of some angiogenesis inhibitors for cancer therapy.

As mentioned above, indirect angiogenesis inhibitors will block the expression or activity of pro-angiogenic proteins like EGFR [80]. For example, Ciardiello et al. evaluated the anti-angiogenic and antitumor activity of gefitinib (ZD1839; Iressa^®^), a small molecule known as an EGFR tyrosine kinase inhibitor (TKI) in human colon (GEO, SW480, and CaCo2), breast (ZR-75-1 and MCF-7 ADR), ovarian (OVCAR-3), and gastric (KATO III and N87) cancer cells, that co-expresses TGF-α and EGFR (pro-angiogenic factor) [81]. Additionally, the U.S. FDA has approved a number of angiogenesis inhibitors for the treatment of cancers (Figure 3, Table 2). R. K. Jain reported that for both direct or indirect anti-angiogenic therapy, the balance between pro-angiogenic and anti-angiogenic factors will be restored through the reduction of vessel permeability and hypoxia and enhancement of the homogeneity of blood flow and perivascular cells coverage [82].

For example, bevacizumab, a recombinant humanized monoclonal antibody to VEGF and known by its brand name, Avastin^®^, blocks tumor cell-derived VEGF-A, impairing the development of new vessels and leading to tumor starvation and, consequently, growth inhibition [83]. It has been observed that the side effects of bevacizumab increased when it was combined with chemotherapy. For example, in the treatment of colorectal cancer treated with IFL (a chemotherapy regimen consisting of concurrent treatment with irinotecan, leucovorin (folinic acid), and fluorouracil) in combination with bevacizumab, bleeding complications were observed [84,85]. Combination therapy of bevacizumab with carboplatin and paclitaxel improved the overall response and time to progression in patients with advanced or recurrent non-small-cell lung cancer, but severe or fatal pulmonary hemorrhage has been observed [86]. In ovarian cancer, the combination of platinum-based chemotherapy with bevacizumab delayed progression and improved survival for newly diagnosed ovarian cancer patients after initial surgery [87].

Thalidomide, with the brand name of Immunoprin, is known for the treatment of multiple myeloma and other types of cancers that express angiogenic cytokines such as VEGF and bFGF [88]. Lenalidomide (Revlimid^®^) is a derivative of thalidomide, and it is employed for the treatment of multiple myeloma and a specific type of myelodysplastic syndrome (MDS) [89].

Sunitinib (previously known as SU11248) is a TKI and is used to treat kidney cancer; it was also approved by the FDA to treat renal cell carcinoma (RCC) and imatinib-resistant gastrointestinal stromal tumors. Sunitinib also showed promising activity in the treatment of other tumors such as neuroendocrine tumors [90], advanced non-small-cell lung cancer [91], breast cancer [92], and colorectal cancer [93]. Sorafenib (Nexavar^®^) is a TKI drug that is approved for the treatment of liver cancer (hepatocellular carcinoma) [94], kidney cancer (advanced renal cell carcinoma) [95], and radioactive iodine-resistant advanced thyroid carcinoma [96].

Temsirolimus (Torisel^®^) was approved by the FDA in 2007 for the treatment of advanced RCC. It is an inhibitor of the mammalian target of rapamycin (mTOR), an enzyme that regulates cell growth and proliferation [97]. By activation of mTOR, c-Myc, and HIF-1α will be stimulated, which results in an increase in genes that promote VEGF-associated angiogenesis, proliferation (cyclin D1), and cell survival (survivin) [98]. Temsirolimus is also known to disrupt angiogenesis, which plays an important role in the development and progression of RCC [99,100,101]. The combination of temsirolimus with vorinostat showed higher anticancer activity compared with temsirolimus alone in both in vitro and in vivo models of RCC. The effectiveness of the combination was due to a decrease of the surviving levels, apoptotic induction, and improved reduction of angiogenesis [100]. Axitinib (Inlyta^®^) is another FDA-approved small molecule TKI shown in clinical trials to induce partial response for the treatment of RCC and several other tumor types. Phase II trials with this agent alone or in combination with chemotherapeutic drugs were reported in several types of malignancy [102]. Additional FDA-approved chemotherapeutic drugs are listed in Table 2.

## 5. Conclusions

Angiogenesis plays a significant role in tumor progression. Effective inhibition of tumor angiogenesis might arrest or halt tumor progression but would not eradicate the tumor as a stand-alone therapy, especially with a single mechanism anti-angiogenic agent. Hence, the combination of an anti-angiogenesis agent and chemotherapy might be essential for effective tumor treatment.

## Figures and Tables

**Table 1:** Selected list of endogenous angiogenesis inhibitors and mechanisms of action.

Endogenous Angiogenesis Inhibitors	Mechanisms	Reference
Soluble VEGF-1	Decoy receptors for VEGF-B	[5]
Angiostatin	Suppress EC adhesion, migration, proliferation	[6]
Thrombospondin-1 and -2	Suppress EC adhesion, migration, proliferation	[7]
Angiopoietin-2	Oppose Angiopoietin 1	[8]
Platelet Factor-4	Inhibit bFGF (FGF2) and VEGF binding	[9,10]
Endostatin	Suppress EC adhesion, migration, proliferation	[6,11]
Anti-thrombin III Fragment	Suppress EC adhesion, migration, proliferation	[12]
Osteopontin	Serve as ligand for integrin binding	[13]
Collagen	Substrate for MMPs	[14,15]
Kininogen Domains	Suppress EC adhesion, migration, proliferation	[16]
Tissue Factor Pathways Inhibitor	Antagonist for Tissue Factor	[17]
Vasostatin	Suppress EC adhesion	[18,19]
Calreticulin	Suppress EC adhesion	[20]
TIMPs	Suppress EC adhesion	[21,22]
A cartilage-derived angiogenesis inhibitor	Suppress EC adhesion	[23]
Meth-1 and Meth-2	Suppress EC adhesion	[24]
Maspin	Inhibits proteases	[25]
Laminin 511	Suppresses metastases	[26,27]
CCN3	Suppresses EC adhesion	[28]
Endorepellin	Suppresses EC adhesion	[29]
MULTIMERIN2 (Endoglyx-1)	Suppresses EC migration	[30]

Abbreviations: VEGF: vascular endothelial growth factor; EC: endothelial cells; FGF: fibroblast growth factor; MMP: matrix metalloproteinase; TIMP: tissue inhibitor of metalloproteinase.

**Table 2:** FDA-approved inhibitors. These anti-angiogenesis strategies are being used in conjunction with other anticancer chemotherapeutics.

Generic Name	FDA-Approved Indication
Bevacizumab	Colorectal, non-small-cell lung, and glioblastoma multiforme
Thalidomide	Myeloma
Lenalidomide	Myeloma (myelodysplastic syndrome (MDS))
Sorafenib	Renal cell and hepatocellular carcinoma
Sunitinib	Renal cell and gastrointestinal carcinoma
Temsirolimus	Renal cell carcinoma
Axitinib	Renal cell carcinoma
Pazopanib	Renal cell carcinoma, kidney cancer, and advanced soft tissue sarcoma
Cabozantinib	Thyroid cancer
Everolimus	Kidney cancer, advanced breast cancer, pancreatic neuroendocrine tumors (PNETs), and subependymal giant cell astrocytoma
Ramucirumab	Stomach cancer and gastroesophageal junction adenocarcinoma
Regorafenib	Colorectal cancer and gastrointestinal stromal tumor
Vandetanib	Thyroid cancer
Ziv-aflibercept	Colorectal cancer
